# Effects of size at birth, childhood growth patterns and growth hormone treatment on leukocyte telomere length

**DOI:** 10.1371/journal.pone.0171825

**Published:** 2017-02-08

**Authors:** Carolina C. J. Smeets, Veryan Codd, Matthew Denniff, Nilesh J. Samani, Anita C. S. Hokken-Koelega

**Affiliations:** 1 Department of Pediatrics, subdivision of Endocrinology, Erasmus University Medical Center, Rotterdam, The Netherlands; 2 Department of Cardiovascular Sciences, University of Leicester, Leicester, United Kingdom; 3 NIHR Leicester Cardiovascular Biomedical Research Unit, Glenfield Hospital, Leicester, United Kingdom; 4 Dutch Growth Research Foundation, Rotterdam, The Netherlands; Shanghai Jiaotong University School of Medicine Xinhua Hospital, CHINA

## Abstract

**Background:**

Small size at birth and rapid growth in early life are associated with increased risk of cardiovascular disease in later life. Short children born small for gestational age (SGA) are treated with growth hormone (GH), inducing catch-up in length. Leukocyte telomere length (LTL) is a marker of biological age and shorter LTL is associated with increased risk of cardiovascular disease.

**Objectives:**

To investigate whether LTL is influenced by birth size, childhood growth and long-term GH treatment.

**Methods:**

We analyzed LTL in 545 young adults with differences in birth size and childhood growth patterns. Previously GH-treated young adults born SGA (SGA-GH) were compared to untreated short SGA (SGA-S), SGA with spontaneous catch-up to a normal body size (SGA-CU), and appropriate for gestational age with a normal body size (AGA-NS). LTL was measured using a quantitative PCR assay.

**Results:**

We found a positive association between birth length and LTL (p = 0.04), and a trend towards a positive association between birth weight and LTL (p = 0.08), after adjustments for gender, age, gestational age and adult body size. Weight gain during infancy and childhood and fat mass percentage were not associated with LTL. Female gender and gestational age were positively associated with LTL, and smoking negatively. After adjustments for gender, age and gestational age, SGA-GH had a similar LTL as SGA-S (p = 0.11), SGA-CU (p = 0.80), and AGA-NS (p = 0.30).

**Conclusions:**

Larger size at birth is positively associated with LTL in young adulthood. Growth patterns during infancy and childhood are not associated with LTL. Previously GH-treated young adults born SGA have similar LTL as untreated short SGA, SGA with spontaneous catch-up and AGA born controls, indicating no adverse effects of GH-induced catch-up in height on LTL.

## Introduction

Small size at birth and catch-up in weight for length in early life are associated with an increased risk for cardiovascular disease (CVD) in later life [1–3]. The mechanisms underlying these associations are not fully understood, but it appears that early life growth trajectories have programming effects on later health outcomes [4,5]. Ten percent of all children born small for gestational age (SGA) show insufficient catch-up growth and remain short [6]. These children can nowadays be treated with growth hormone (GH) from the age of four years, resulting in a significant catch-up in length [7]. GH treatment has several positive effects on metabolic health, but the long-term effects on later life health are less known [8]. Previous studies have suggested that shorter, smaller bodies have advantages in terms of health and longevity [9,10].

Telomeres are noncoding repeating DNA sequences at the end of each chromosome. Their primary function is to maintain genomic stability [11,12]. Telomeres shorten with each cell division due to the inability of DNA polymerase to fully replicate the end of the chromosome. When telomeres are reduced to a critical length, the cell enters a state of arrest [13]. Since leukocyte telomere length (LTL) declines with increasing age, it can serve as an index for biological aging. LTL is influenced by oxidative and replicative stress, and shorter LTL is associated with increased risk for CVD [14,15].

In this study, we investigated whether size at birth, growth patterns during infancy and childhood, and GH treatment influence LTL of young adults. We hypothesized that small size at birth and accelerated weight gain during infancy lead to shorter LTL. We also hypothesized that the gradual catch-up in length caused by GH treatment does not lead to increased attrition of telomeres and thus does not influence LTL. To address the fact that those born SGA have an already increased risk for CVD, we compared the data of previously GH-treated young adults born SGA with untreated short young adults born SGA. To study whether GH-induced catch-up growth has a similar effect on LTL as spontaneous catch-up after SGA birth, we also compared the GH-treated group to a group of young adults born SGA with spontaneous catch-up (SGA-CU).

## Methods

### Subjects

The total population consisted of 545 participants: 470 healthy participants from the PROGRAM and PREMS study cohorts [2,16], and 75 age-matched participants who had participated in a GH trial [8,17]. The 470 healthy participants fulfilled the same inclusion criteria: 1) age 17–24 yr; 2) born singleton; 3) Caucasian; 4) uncomplicated neonatal period without severe asphyxia (defined as an Apgar score below three after five minutes), sepsis, or long-term complications of respiratory ventilation and/or oxygen supply. Participants were randomly selected from hospitals in the Netherlands, where they had been registered because of their small size at birth (birth length <-2 standard deviation score (SDS)), short stature (adult height <-2 SDS) or being born preterm (gestational age <36 weeks). Young adults born appropriate for gestational age (AGA) were asked to participate via advertisements at schools with different educational levels. For the last analysis, we additionally included the 75 GH-treated subjects. All subjects were born SGA (birth weight and/or birth length <-2 SDS) and received GH treatment during childhood because of their short stature, for ≥7 years. All participants received biosynthetic GH at a dose of 1 mg/m^2^/day (0.035 mg/kg/d), sc at bedtime. Every three months, GH dose was adjusted to the calculated body surface area. The data of the GH group were compared to those of three subgroups based on their size at birth and their adult stature: untreated young adults born SGA (birth length <-2 SDS) with persistent short stature (adult height <-2 SDS) (SGA-S, n = 48); young adults born SGA (birth length <-2 SDS) with spontaneous catch-up growth resulting in a normal adult height (>-1 SDS) (SGA-CU, n = 89); and young adults born appropriate for gestational age with a normal adult height (>-1 SDS) (AGA-NS, n = 135). In order to increase the statistical power for subgroup comparison, the cut-off values for small birth size and short adult height were set at <-2 SDS, and the cut-off-values for normal birth size and normal adult height were set at >-1 SDS.

This study was conducted according to the Helsinki Declaration. The Medical Ethics Committee of Erasmus Medical Centre approved the study. Written informed consent was obtained from all participants and/or their parents.

### Measurements

Birth data were obtained from hospital records, primary health care records and general practitioner records. Height was measured to the nearest 0.1 cm by a Harpenden stadiometer, weight to the nearest 0.1 kg by a scale (Servo Balance KA-20-150S). All anthropometric measurements were performed by a trained investigator, according to standardized methods. The measurements were performed twice, the mean value was used for analyses. Fat mass, fat mass percentage and lean body mass were measured on one Dual-energy X-ray Absorptiometry (DXA) machine (Lunar Prodigy, GE Healthcare, Chalfont St Giles, England) [18]. Quality control was performed daily. Information regarding socioeconomic status (SES) based on education level, and smoking of the participants was obtained using questionnaires [19].

### LTL assessment

Genomic DNA was isolated from peripheral leukocytes using standard procedures. All LTL measurements were performed in the laboratory of the University of Leicester, using the quantitative PCR-based technique as previously described [20]. Telomere sequence copy number (T) was compared with a single copy gene number in the genome 36B4 (S) and telomere length expressed as a T/S ratio. All T and S values were calculated relative to a calibrator DNA (genomic DNA from the K562 cell line) that was included on every plate, minimizing the potential for inter-run variation. All samples were checked for concordance between duplicate values. Samples showing a difference of greater than 0.2 cycles in the take-off value or amplifying outside of the linear range of the assay were excluded and re-run. Reproducibility of the assay was tested by re-running samples on separate days. The mean inter-run coefficient of variation for the T/S ratio was 3.33%.

### Calculations and statistical analysis

Standard deviation scores (SDS) for birth length and birth weight were calculated in order to correct for gender and gestational age, and SDS for adult height and weight were calculated to correct for gender and age [21], all using the growth analyser software (http://www.growthanalyser.org). Fat mass percentage SDS was calculated according to age- and sex-matched Dutch reference values [22]. Because lean body mass is strongly related to height, lean body mass was expressed as SDS for height and sex [22].

Means and SD were used to describe the distribution of continuous variables. Multiple linear regression analyses were performed to determine whether size at birth (i.e. birth length and birth weight) and childhood growth patterns (i.e. the degree of catch-up in length and weight from birth to adulthood) were significant predictors of LTL. Because of collinearity between birth weight and birth length, these variables were analyzed in separate models. Adjustments were made for age and gender, and additionally for gestational age, body composition, smoking and SES. Because the study group had been selected on birth length and adult height, the interaction term birth length SDS*adult height SDS was added to the analysis, in order to ensure that the effect of these variables was modeled correctly.

Quartiles of weight gain during the first 12 months of life were determined in the total group, except for the GH-treated subjects and for men and women separately. ANCOVA was used to determine differences in LTL between the lowest and highest quartiles, corrected for age and gestational age. Lastly, we analyzed whether there were differences in LTL between the SGA-GH subgroup and the SGA-S, SGA-CU and AGA subgroups. In this analysis, we additionally adjusted for age, gender and gestational age. Results were considered statistically significant if the p-value was <0.05. Statistical package SPSS version 21.0 (SPSS, Inc., Chicago, IL) was used for all analyses.

## Results

Clinical characteristics of the total study population (n = 470), and for men and women separately are shown in Table 1. Mean (SD) age of the total population was 20.9 (1.7) years. Mean (SD) lean body mass SDS was higher in men than in women (-0.12 (1.1) versus -0.62 (1.3), resp.; p<0.001). The other clinical characteristics were similar in men and women. Mean (SD) LTL was shorter in men than in women (p = 0.02).

**Table 1:** Clinical characteristics.

	Total group (n = 470)	Men (n = 204)	Women (n = 266)	p-value
Age (yrs)	20.9 (1.7)	21.0 (1.7)	20.8 (1.7)	0.29
Gestational age (wks)	36.7 (3.9)	36.3 (4.0)	37.0 (3.7)	0.06
Birth weight SDS	-0.97 (1.6)	-0.83 (1.7)	-1.07 (1.5)	0.10
Birth length SDS	-1.43 (1.6)	-1.33 (1.6)	-1.52 (1.6)	0.22
Adult height SDS	-0.85 (1.3)	-0.86 (1.2)	-0.85 (1.3)	0.94
Adult weight SDS	-0.52 (1.4)	-0.57 (1.3)	-0.49 (1.4)	0.55
BMI	22.5 (3.7)	22.3 (3.2)	22.6 (4.0)	0.36
Fat mass % SDS	0.62 (0.9)	0.63 (0.9)	0.62 (0.9)	0.89
Lean body mass SDS	-0.41 (1.2)	-0.12 (1.1)	-0.62 (1.3)	**<0.001**
Smoking (%)	27.5	29.5	25.9	0.39
SES (%)				
1	12.4	13.5	11.7	
2	26.6	27.5	26.0	0.78
3	60.9	59.1	62.3	
LTL	3.20 (0.5)	3.14 (0.4)	3.24 (0.5)	**0.02**

Values are given as means (SD). P-values <0.05 are shown in bold. BMI = body mass index; LTL = Leukocyte Telomere Length (in T/S ratio); SES = socioeconomic status

### Factors associated with LTL in the total group

We analyzed the effects of size at birth and postnatal growth on LTL in a multiple regression analysis. First, birth length was analyzed (Table 2). As expected, female gender was positively associated with LTL (p = 0.004). Age was not a significant confounder of LTL in our analyses, probably because of the fact that most subjects in our study population had approximately the same age. In Model A, birth length SDS did not predict LTL (p = 0.36). In Model B, we added gestational age to the model, which proved to be a significant predictor of LTL (β = 0.02, p = 0.002). In Model C, we added adult height and the interaction term birth length SDS*adult height SDS to the model. In this model, there was a trend towards a positive relation between birth length and LTL (β = 0.03, p = 0.06). Adult height was not associated with LTL, and since birth length was included in the model, this shows us that gain in height from birth to adulthood does not predict LTL. Then, parameters of body composition were added to the model (Model D), showing a trend towards a positive relation between LBM SDS and LTL (β = 0.04, p = 0.06). The relation between birth length and LTL was still close to significant in this model (p = 0.08). Finally, we included the possible confounders smoking and SES in the model (Model E), showing an inverse association between smoking and LTL (ß -0.12, p = 0.03). In this last model, gender, gestational age and birth length SDS were all positive predictors of LTL.

**Table 2:** Multiple regression analysis for variables associated with leukocyte telomere length at 21 years of age—Analysis including birth length.

	Model A		Model B		Model C		Model D		Model E	
Variables	ß	p	ß	p	ß	p	ß	p	ß	p
Female gender	0.13	**0.004**	0.12	**0.009**	0.12	**0.006**	0.16	**0.001**	0.15	**0.002**
Age	0.00	0.85	0.00	0.77	0.00	0.80	0.00	0.79	-0.00	0.97
Birth length SDS	0.01	0.53	0.01	0.36	0.03	0.06	0.03	0.08	0.04	**0.04**
Gestational age			0.02	**0.002**	0.02	**0.02**	0.01	**0.048**	0.02	**0.02**
Adult height SDS					-0.02	0.30	-0.02	0.49	-0.03	0.26
Fat mass % SDS							-0.02	0.49	-0.01	0.68
Lean body mass SDS							0.04	0.06	0.03	0.22
SES									0.00	0.99
Smoking									-0.12	**0.03**
Overall p-value	**0.03**		**0.001**		**<0.001**		**<0.001**		**<0.001**	
R^2^ adjusted	0.01		0.03		0.04		0.05		0.07	

ß = regression coefficient. A positive value indicates that the dependent variable LTL will increase with that amount for every unit increase of the independent variable. All analyses where adult height was included were additionally adjusted for the interaction term birth length*adult height SDS. P-values <0.05 are shown in bold.

SDS = standard deviation score; SES = Socioeconomic status (Lowest socioeconomic status is used as the reference for SES analyses).

The same models were used to analyze the relation between birth weight and LTL (Table 3). Instead of height SDS, we included weight SDS to the model, to analyze the influence of weight gain from birth to adulthood. Regarding the variables gender, age, gestational age, LBM and smoking, this analysis showed similar results, except that LBM was a significant predictor of LTL. There was a (trend towards a) significant positive relation between birth weight and LTL in all models. Weight SDS was not a significant predictor of LTL. Since birth weight was included in this model, this shows us that weight gain from birth to adulthood does not predict LTL. Due to the high collinearity between weight and FM%, weight was excluded in Model D. In the final model, there was still a trend towards a positive relation between birth weight SDS and LTL (ß = 0.02, p = 0.08).

**Table 3:** Multiple regression analysis for variables associated with leukocyte telomere length at 21 years of age—Analysis including birth weight.

	Model A		Model B		Model C		Model D		Model E	
Variables	ß	p	ß	p	ß	p	ß	p	ß	p
Female gender	0.11	**0.01**	0.12	**0.03**	0.10	**0.03**	0.15	**0.001**	0.14	**0.004**
Age	0.00	0.96	0.00	0.92	0.00	0.96	0.00	0.85	-0.01	0.89
Birth weight SDS	0.02	0.11	0.01	**0.03**	0.03	0.07	0.03	0.06	0.02	0.08
Gestational age			0.02	**0.004**	0.02	**0.002**	0.01	**0.02**	0.02	**0.003**
Weight SDS					-0.02	0.70				
Fat mass % SDS							-0.01	0.71	0.06	0.86
Lean body mass SDS							0.04	**0.02**	0.07	**0.048**
SES									-0.02	0.76
Smoking									-0.12	**0.03**
Overall p-value	**0.04**		**0.002**		**<0.001**		**<0.001**		**<0.001**	
R^2^ adjusted	0.01		0.03		0.04		0.04		0.05	

ß = regression coefficient. A positive value indicates that the dependent variable LTL will increase with that amount for every unit increase of the independent variable. P-values <0.05 are shown in bold.

SDS = standard deviation score; SES = Socioeconomic status (Lowest socioeconomic status is used as the reference for SES analyses).

### Effects of weight gain and fat mass accumulation during infancy on LTL

**Fig 1:**
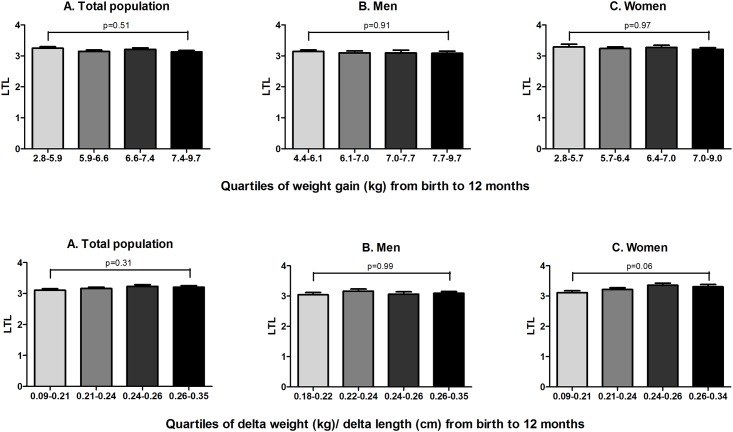
Weight gain and fat mass accumulation during infancy and LTL. Values are given as estimated means ± SEM, adjusted for age and gestational age.

We analyzed the effect of weight gain and fat mass accumulation during the first 12 months of life on LTL. We found no significant correlation between weight gain in the first 12 months of life and LTL (*r* = -0.08, p = 0.15). Subsequently, we stratified the population into quartiles based on weight gain in kg and Δweight (kg)/Δlength (cm) during the first 12 months of life. Fig 1 shows LTL of the total group and men and women separately, adjusted for age and gestational age.

The weight gain analyses showed no difference in LTL between the lowest and highest quartile of weight gain during the first 12 months of life in the total population (p = 0.51), in men (p = 0.91) and women (p = 0.97). The analyses for Δweight/Δlength during the first 12 months of life neither showed significant differences in LTL between the lowest and highest quartile in the total population (p = 0.31) and in men (p = 0.99), and there was a trend towards longer LTL in women with the highest Δweight/Δlength (p = 0.06), indicating no negative effect of fat mass accumulation during the first 12 months of life on LTL in early adulthood.

### Effects of growth hormone treatment on LTL

The effect of long-term GH treatment on LTL was analyzed by comparing LTL between subjects born SGA and treated with GH (SGA-GH) versus age-matched untreated short subjects born SGA (SGA-S), subjects born SGA with spontaneous catch-up during childhood (SGA-CU), and AGA born controls with a normal adult stature (AGA-NS). Clinical characteristics of the subgroups are shown in Table 4. There were significant differences in gender, age, gestational age, birth length SDS, birth weight SDS, adult height SDS, weight SDS, BMI, body composition and distribution of SES between the groups.

**Table 4:** Clinical characteristics of the subgroups.

	SGA-GH (n = 75)	SGA-S (n = 48)	SGA-CU (n = 89)	AGA-NS (n = 135)
Male/female	42/33^1^^,^^2^	16/32	35/54	64/71
Age (yrs)	20.2 (2.4)^2^	20.8 (1.8)	20.9 (1.6)	20.8 (1.7)
Gestational age (wks)	36.2 (4.0)^1^	38.2 (3.1)	36.3 (3.2)	36.3 (4.0)
Birth weight SDS	-2.44 (1.2)^3^	-2.07 (0.9)	-2.31 (0.8)	0.29 (1.3)
Birth length SDS	-3.42 (1.5)^2^^,^^3^	-3.05 (0.9)	-2.93 (0.8)	0.22 (0.8)
Adult height SDS	-1.42 (0.8)^1^^,^^2^^,^^3^	-2.55 (0.5)	-0.17 (0.6)	0.18 (0.8)
Adult weight SDS	-1.01 (1.3)^2^^,^^3^	-1.44 (1.5)	0.08 (1.2)	0.09 (1.0)
BMI	20.5 (2.7)^1^^,^^2^^,^^3^	23.3 (4.4)	22.8 (4.3)	22.3 (3.1)
Fat mass % SDS	0.88 (0.9)^1^	1.60 (0.8)	1.19 (0.8)	0.97 (0.8)
Lean body mass SDS	-0.68 (1.3)^1^	0.09 (1.6)	-0.72 (1.1)	-0.63 (1.0)
Smoking (%)	28.6	25.0	29.4	24.1
SES (%)				
1	9.1	20.9	16.4	3.4
2	63.6^3^	30.2	31.5	17.8
3	27.3	48.8	52.1	78.8
LTL	3.12 (0.5)	3.30 (0.4)	3.07 (0.4)	3.20 (0.5)

Values are given as means (SD).

^1^ p<0.05 compared to SGA-S.

^2^ p<0.05 compared to SGA-CU.

^3^ = p<0.05 compared to AGA-NS.

BMI = body mass index; LTL = Leukocyte Telomere Length (in T/S ratio); SES = socioeconomic status; SGA-GH = birth length <-2 SDS, treated with growth hormone; SGA-S = birth length <-2 SDS, adult height <-2 SDS; SGA-CU = birth length <-2 SDS, with spontaneous catch-up to adult height >-1 SDS; AGA-NS = birth length >-1 SDS, adult height >-1 SDS

**Fig 2:**
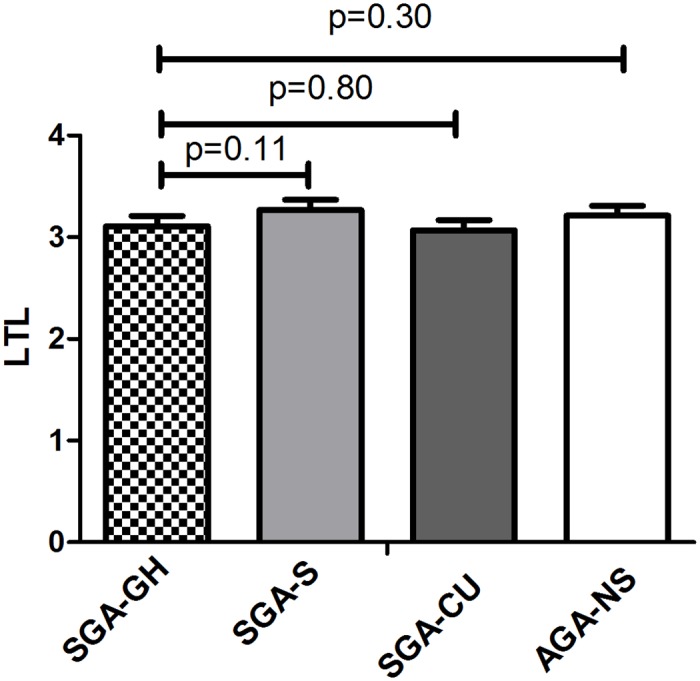
Comparison of LTL in the subgroups. Values are given as estimated means ± SEM, adjusted for gender, age and gestational age. SGA-GH = birth length <-2 SDS, treated with growth hormone; SGA-S = birth length <-2 SDS, adult height <-2 SDS; SGA-CU = birth length <-2 SDS, with spontaneous catch-up to adult height >-1 SDS; AGA-NS = birth length >-1 SDS, adult height >-1 SDS

Fig 2 shows estimated mean (SE) LTL of the subgroups, adjusted for gender, age and gestational age. The SGA-GH subgroup had a similar LTL as the SGA-S group (p = 0.11), the SGA-CU group (p = 0.80) and the AGA-NS group (p = 0.30).

## Discussion

We found a positive association between birth length and LTL, and a trend towards a positive association between birth weight and LTL. No associations were found between gain in weight for length during infancy and childhood and adult body size, and no influence of GH-induced catch-up growth on LTL.

We performed a multiple regression analysis in the total group to analyze the effects of size at birth, adult body size and weight gain during childhood on LTL. Birth length was positively associated with LTL, and there was a trend towards a positive association between birth weight and LTL. These associations were adjusted for possible confounders, such as gender and gestational age, indicating an independent effect of size at birth on LTL. Since previous reports have shown that small size at birth is associated with risk for CVD in later life, it could be that LTL is one of the links between birth size and later life CVD-risk. Our results are in concordance with a recent study of de Zegher et al., showing that telomere lengths are shorter in SGA newborns than in AGA newborns [23]. On the other hand, a study of Kajantie et al. found no correlation between size at birth and LTL [24]. Future studies should aim at exploring the possible underlying mechanisms of the association between size at birth and LTL, such as increased oxidative stress in those born after intra-uterine growth restriction.

We found a positive association between gestational age and LTL. In a previous report we have shown that those born preterm have shorter LTL than those born at term [25], which could be due to increased oxidative stress in those born preterm. Our findings that female gender is positively associated with LTL, and smoking negatively, also correspond to previous studies [26,27], although it is striking that smoking already influences LTL at such a young age.

In our study, extensive data on adult body composition were available: next to weight SDS and BMI, we also measured fat mass and lean body mass using DXA. We found no relation between fat mass percentage and LTL. This result is in contrast with previous studies, showing that obesity is associated with shorter LTL in both children and adults, probably due to the fact that obesity causes increased oxidative stress, which exacerbates telomere attrition [28,29]. The main difference with our study is that these studies compared groups with a high BMI to groups with a normal BMI, while we modelled the effect of the continuous variables weight SDS, fat mass and lean body mass in a multiple regression analysis. It could be that the relation between obesity and shorter LTL is subtle, and therefore not present in our group of healthy young adults, with a low percentage of obese participants.

Gain in weight, gain in height and fat mass accumulation from birth to adulthood were not associated with LTL in our study. We previously showed that growth patterns during infancy have programming effects on health in later life [2,30]. We, therefore, additionally analyzed whether catch-up in weight and fat mass accumulation during the first year of life were associated with LTL. This analysis showed no difference in LTL between those in the lowest and highest quartile of weight gain and fat mass accumulation during the first year of life. Our results are in contrast with a recent study, that also measured LTL by quantitative PCR, and found an inverse association between weight gain in the first 12 months and LTL at the age of 70 [31]. This association was only found in women. Based on these results, the investigators suggested that rapid growth during the perinatal period accelerates cellular aging in late adulthood. The main differences with our study are that the effects of Δweight/Δlength as a proxy of fat mass accumulation were not tested in that study and the fact that the participants were much older. It would, therefore, be interesting to analyze whether the association between weight gain during infancy and LTL becomes significant at a later age, when age-associated diseases also become more apparent.

To our knowledge, we are the first to evaluate whether GH treatment has an effect on LTL. Young adults born SGA who were treated with GH during childhood had similar LTL as age-matched untreated short subjects born SGA, subjects born SGA with spontaneous catch-up and controls born AGA with a normal stature. Thus, GH-induced catch-up in length does not lead to shorter LTL in young adults born SGA. It seems that a gradual catch-up in length, after the age of four years (when GH therapy is usually started), does not lead to increased replicative stress. Since data on age-associated diseases, such as CVD, long after cessation of GH treatment are scarce, this result is reassuring [32]. Our results support previous data, showing that there are no adverse effects of long-term GH treatment on CVD-risk and that adults who were treated with GH during childhood do not have increased mortality rates [33–35].

Although the multiple regression analyses resulted in significant associations between multiple variables and LTL, it should be noted that the R^2^ was small in all analyses, indicating that there are other determinants of LTL, that were not included in our analyses.

As telomere length measured in different tissues of the same patient, are highly correlated [36,37], LTL not only mirrors the aging process in circulating immune cells, but in other tissues as well. This way, LTL might reflect the vulnerability of our cells to exogenous stress factors in general. The mechanisms underlying telomere shortening are complex. It is known that, next to genetic factors, oxidative and replicative stress are main determinants of LTL. However, the generalizability of shorter telomere length due to replicative stress to other cells is not well studied. For future studies, it would be interesting to measure telomere length in other tissues as well, to see whether low birth weight and subsequent catch-up growth influence telomere length in other tissues that might be more prone to replicative stress (for example bone and muscle tissue).

One of the main strengths of the present study is the large group of young adults that was included, with a great variation in size at birth and childhood growth patterns. Because we oversampled subjects with extreme variants of normal growth, such as subjects born SGA with and without catch-up growth, we created greater contrast in the study population, which contributed to more statistical power.

In conclusion, we found that size at birth, gestational age and female gender are positively associated with LTL and smoking negatively, while adult fat mass and gain in weight and height from birth to adulthood and during infancy were not associated with LTL. Young SGA adults who received GH treatment during childhood have similar LTL as age-matched untreated short SGA, SGA with spontaneous catch-up and controls born AGA, indicating no adverse effects of GH treatment on LTL, which is reassuring.

## Abbreviations

AGA: Appropriate for gestational age
CVD: Cardiovascular disease
GH: Growth Hormone
LTL: Leukocyte Telomere Length
PCR: Polymerase Chain Reaction
SGA: Small for Gestational Age
SES: Socio-economic status
T/S ratio: Telomere to single-gene copy ratio
